# Supplementary figures and images for: Active School Transport among Children from Canada, Colombia, Finland, South Africa, and the United States: A Tale of Two Journeys
Source: Int J Environ Res Public Health. 2020 May 28;17(11):3847. doi: 10.3390/ijerph17113847 (PMC7312928; doi:10.3390/ijerph17113847)

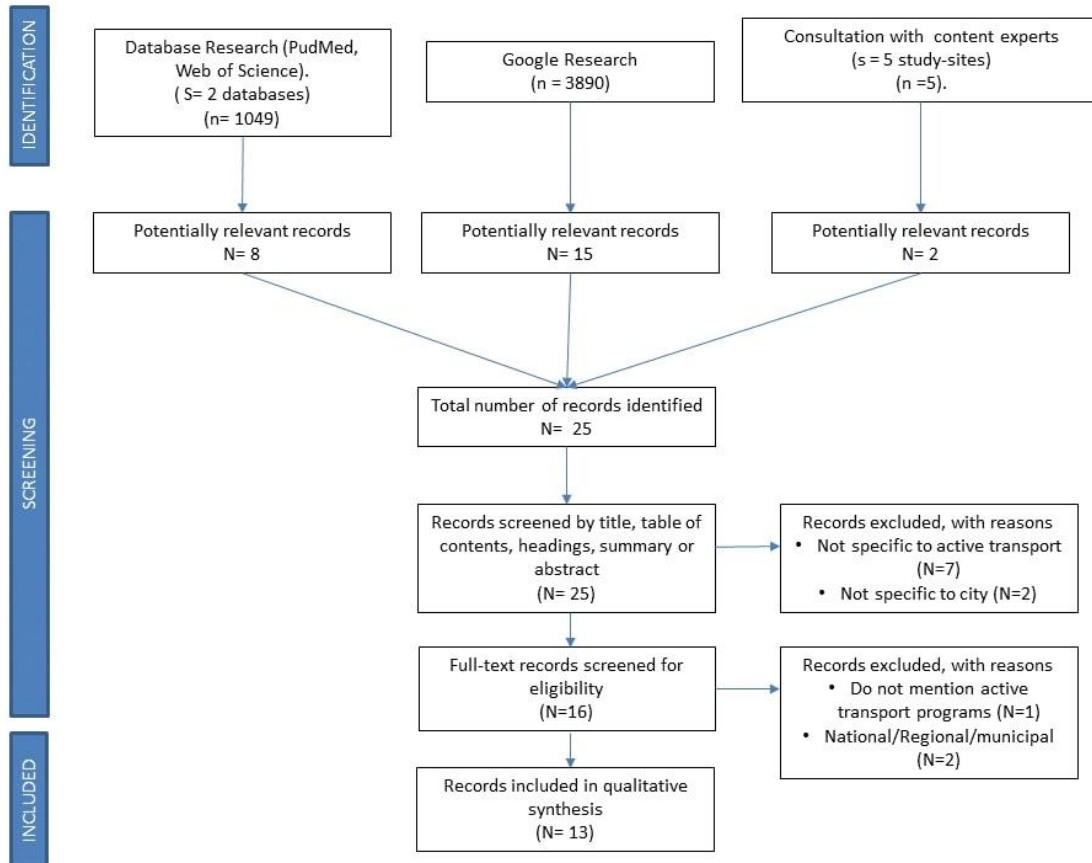

**Figure S1.** Policy search flow chart.

Supplement: Supplementary file 1 [file ijerph-17-03847-s001.pdf]
